# Seasonal Variation of Essential Oils Composition in a Genetically Diverse Population of *Lippia alba* and Its Influence on Antibacterial Activity

**DOI:** 10.1002/cbdv.71745

**Published:** 2026-10-06

**Authors:** Marina Erê Almeida Hummel Pimenta Santos, Taila Gomes Botelho, Camila Delarmelina, Walter José Siqueira, Marta Cristina Teixeira Duarte, Marcia Ortiz Mayo Marques, Jorge Maurício Costa Mondego

**Affiliations:** ^1^ Centro De P&D De Recursos Genéticos Vegetais Instituto Agronômico (IAC) Campinas Sao Paulo Brazil; ^2^ Pós‐Graduação Em Agricultura Tropical e Subtropical Instituto Agronômico (IAC) Campinas Sao Paulo Brazil; ^3^ Centro Pluridisciplinar de Pesquisas Químicas, Biológicas e Agrícolas (CPQBA) Universidade Estadual de Campinas (UNICAMP) Paulínia Sao Paulo Brazil

**Keywords:** antimicrobial activity, chemotype, essential oils, *Lippia alba*, seasonal variation

## Abstract

*Lippia alba* is an aromatic plant whose essential oils (EOs) have been characterized as promising antimicrobial agents. It has been established that *L. alba* chemotypes are influenced by both genetic and environmental factors. We evaluated the EO yield, composition, and antimicrobial activity of 20 genotypes from a sympatric genetically diverse population of *L. alba*. These analyses were performed over two seasonal periods, during which climate data were collected and leaf nutrients assessed. EO yield was highest on average during summer, although genotype‐specific patterns were observed. Gas chromatography–mass spectrometry identified 47 compounds, which 27 were observed to be shared among the 20 genotypes. A total of 10 of the 20 genotypes studied had seasonal shifts in predominant constituents. Minimum inhibitory concentration (MIC) assays showed high antimicrobial activity in summer samples, followed by spring, despite being more effective in the first seasonal data. Micronutrients data suggest that nutrient variation may be associated with changes in essential oil composition. Though exploratory, these results highlight the importance of considering both genotype and harvest season for EO standardization in antimicrobial phytotherapy and other potential applications.

## Graphical abstract

The essential oils profile, antibacterial activity and leaf nutrients contents of a *Lippia alba* genetically diverse population are examined regarding seasonal harvesting.

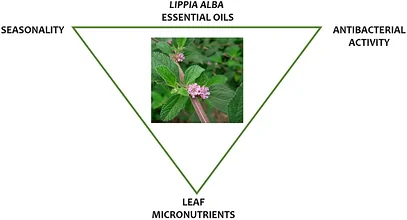

## 1 Introduction

Growing concerns about antimicrobial resistance have stimulated the search for therapeutic alternatives based on natural products, especially compounds derived from aromatic plants. These compounds possess multiple targets of action and a lower propensity for the development of bacterial resistance [1]. Essential oils (EOs) are plant‐derived complex mixtures comprising monoterpenes, sesquiterpenes, and phenylpropanoids, among other classes of substances, which exhibit broad antimicrobial activity against Gram‐positive and Gram‐negative bacteria [2]. Among these microorganisms, *Escherichia coli* and *Staphylococcus aureus* stand out for their ability to form biofilms, survive in adverse environmental conditions, and develop multiple resistance mechanisms. Combating these mechanisms requires compounds capable of destabilizing membranes, modulating regulatory genes, and affecting intracellular targets [2, 3]. Recent studies demonstrate that EOs with distinct chemical profiles act primarily by disrupting the bacterial lipid bilayer membrane, causing loss of membrane integrity and leakage of cytoplasmic content, which reinforces their potential for innovative antimicrobial applications [4].

*Lippia alba* (Mill.) N. E. Brown is an aromatic plant commonly known as “erva‐cidreira‐do‐campo” (field lemon balm) or “falsa‐melissa” (false lemon balm). It belongs to the Verbenaceae family and has emerged as one of the most promising species for the development of natural antimicrobial agents [5]. *L. alba* exhibits a wide diversity of chemotypes, largely influenced by genetic factors, especially the ploidy of specimens genotypes. These comprise diploid (2*n* = 30), triploid, tetraploid (2*n* − 60), and hexaploid (2*n* = 90) cytotypes, each associated with distinct morphological, physiological, and biochemical characteristics [6]. Several studies provide data on the evolutionary dynamics of the relationship between genome distribution and chemical diversity. For instance, the citral dominant chemotype, often exceeding 70% of the EO content, occurs preferentially in diploid and tetraploid accessions [7]. In contrast, linalool dominant chemotypes are predominantly triploids and represent an early diverging lineage suggesting that linalool dominance is an ancestral chemotype [6, 7]. Although there is a strong correlation between specific chemotypes and ploidy levels, evidence suggests that distinct chemotypes can occur within the same polyploid group [8]. Thus, these critical observations move beyond a simplistic model of ploidy‐driven chemistry, arguing instead that the distribution of chemotypes in *L. alba* is the product of a dynamic interplay between polyploidization‐driven evolution and ecological or biochemical selection acting on specific metabolic pathways. Together, these factors shape the remarkable chemical polymorphism diversity observed in this species complex contributing to its adaptive potential.

Environmental factors also contribute to chemotype plasticity in EO profiles and yields. For instance, location, seasonality, light availability, and nutritional levels can alter the relationship between genome size and composition, influencing the expression of genes involved in terpene production ([9]; Lopes et al. 10). The modulation of EOs chemical composition of by seasonality has been highlighted as one of the most important environmental factors in defining the metabolic profile of tropical aromatic species [11]. Although chemotypes (ex. citral or linalool) remains stable, the quantitative proportions of individual terpenes within a chemotype can be influenced, potentially leading to discernible variations (Lopes et al. 10). Among environmental factors, nutrients play an essential role in metabolism, directly influencing the growth, development, and production of secondary metabolites like terpenoids [12]. Macronutrients such as nitrogen (N), phosphorus (P), and potassium (K), along with micronutrients like iron (Fe), zinc (Zn), and magnesium (Mg) are essential for enzymatic activities and structural components involved in terpene biosynthesis [12]. Depletion of macronutrients like phosphorus in *Melissa offinalis* L. (lemon balm) significantly alters chemical composition primarily affecting the citral ratio [13]. In contrast, in *Achillea millefolium* reduced phosphorus increases EO yield by 71% [14]. Therefore, analyzing leaf nutrient status provides a comprehensive approach to precise nutritional management aiming to maximize the quality and chemical stability of EOs in aromatic plants.

Microbiological assays reveal that EOs rich in citral and linalool tend to exhibit greater efficacy against *S. aureus* and *E. coli*, reinforcing the direct influence of chemical composition on antimicrobial action [2]. Systematic reviews also indicate that minimum inhibitory concentration (MIC) values for different *L. alba* chemotypes vary widely but frequently fall within ranges consistent with bacteriostatic and bactericidal activities relevant to pharmaceutical and food industries [9]. This compositional variation directly influences the effectiveness of EOs against biofilm‐forming bacteria, as small changes in the proportions of monoterpenes can profoundly alter the ability to disrupt membranes, inhibit the extracellular matrix, and interfere with virulence genes. The interaction between EOs and antibiotics can potentiate the action of conventional drugs against *S. aureus*, reducing MICs and affecting resistance mechanisms [15]. In *E. coli*, EOs such as citral increase cell membrane permeabilization [16, 17]. Comparative studies between dry and rainy seasons also demonstrate that EOs with higher levels of oxygenated monoterpenes tend to exhibit better antibacterial performance [18], underscoring the need to consider seasonality in the production and standardization of essential oils intended for antimicrobial use.

Our study aims to investigate how seasonality modulates the chemical composition of a sympatric genetically diverse population of *L. alba* and to assess whether these changes influence antimicrobial activity against *E. coli* and *S. aureus*.

## 2 Materials and Methods

### 2.1 Plant Material

Experiments were performed using *L. alba* plants from a genetically diverse population maintained at the germplasm bank of the Agronomic Institute of Campinas (IAC, Campinas, Brazil; 22°53′S, 47°5′W, 664 m a.s.l.). This research was registered with SisGen in compliance with the request of the Ministry of the Environment of Brazil (SisGen/MMA: A9C8639). The location weather is classified as a humid subtropical climate (Cwa) with dry winters according to the Köppen–Geiger classification, experiencing distinct rainy summers with specific conditions corresponding to the seasonal yield. The soil at germplasm site is an Eutric Ferritic Red Latosol (Typic Haplustox).

This germplasm collection comprised a selection of morphologically distinct plants of *L. alba*, collected across various locations in Brazil, exhibiting distinct chemical profiles (Ming et al., 5). The selected accessions were individually staked for multiplication in experimental beds at the Institute, and subsequently, hybridization crosses were carried out in an open pollination field [5, 19]. Seeds obtained from open‐cross fruits were selected and germinated in small containers containing garden soil and coarse sand. The seedlings were transferred to the open field in March 2011. Out of the 100 genotypes available in the germplasm bank, 20 with distinct chemical profiles were selected based on previous studies by the same research group [19, 20, 21] (Additional Table 1).

Initial harvests were carried out across two seasons according to Southern hemisphere: summer 2021 (Su‐21; January 25) and spring 2021 (Sp‐21; October 27). Subsequent experiments throughout 2022 revealed that antimicrobial activities varied by harvest period in certain genotypes. Based on these findings, two additional harvests were conducted in autumn 2023 (Au‐23; April 24) and winter 2023 (Wi‐23; July 25). To evaluate a complete seasonal cycle, further harvests were executed during autumn 2024 (Au‐24; May 21), winter 2024 (Wi‐24; August 20), spring 2024 (Sp‐24; November 26), and summer 2025 (Su‐25; February 21). All harvests were performed between 08:00 and 11:00. The evaluated plants were of uniform age, ranging from 10 years old in 2021 to 14 years old in 2025. Throughout the experimental period, the plants were neither pruned nor treated with pesticides or fertilizers. Irrigation was applied once per week, and harvests occurred 3 to 5 days’ post‐irrigation. The *L. alba* germplasm collection was monitored weekly for pathogens and pests, with no progression of disease or insect infestation observed. Flowering occurred during the spring and summer seasons. Although multiple clonal replicates were available for some genotypes, the majority of genotypes in the germplasm collection were represented by a single plant. Consequently, one individual specimen was selected per genotype to ensure consistency across the study.

Representative leafy branches of each genotype were harvested. Leaves were healthy with no visible disease or nutritional deficit. Branches were dried in a greenhouse at room temperature for 3 weeks. After drying, leaves were manually separated from the branches and crushed. A 50 g aliquot of dry leaves was weighed for essential oil (EO) extraction via hydrodistillation in a Clevenger apparatus for 2 h. The resulting EOs were transferred into sterile amber glass vials (5 mL) and stored in the dark under refrigeration at −20°C.

### 2.2 Extraction and Evaluation of Chemical Composition of Essential Oils

The essential oil samples were diluted in ethyl acetate (Tedia, chromatographic grade, Fairfield, OH, USA, 1.0 mg mL^−1^), and 1 µL of the solution was injected. The analysis was performed using a Thermo Scientific gas chromatograph (model TRACE 1300 Series GC) coupled to a mass spectrometer (model ISQ 7000) and a Triplus RSH autosampler. The injector was maintained at 220°C, with a split ratio of 1:20 and a carrier gas (helium, 99.9999% purity) flow rate of 1.0 mL min^−1^. The mass spectrometer (MS) operated in full scan mode by electron ionization (70 eV) with an acquisition range of *m*/*z* 40 to 450. The ion source and MS transfer line were kept at 220°C and 250°C, respectively. The substances were separated using an Rtx‐5 MS capillary column (30 m × 0.25 mm × 0.25 µm) with the following temperature program: 60°C–240°C, at 3°C.min^−1^. Chromeleon software (Thermo Scientific‐Waltham, MA, USA) was used for data acquisition and processing. The compounds were identified by comparative analysis of the mass spectra with the National Institute of Standards and Technology (NIST 14); Flavour & Fragrance Natural & Synthetic Compounds (FFNSC3) libraries, as well as by comparing the linear reteon indices of the substances (LRI) with the literature (Adams, 2017). LRIs were obtained by injecting a series of *n*‐alkanes (C_8_‐C_20_, Sigma‐Aldrich, 99%) under the same chromatographic conditions as the samples, applying the Van den Dool and Kratz (1963) equation [22]. The area normalization method was applied to obtain relative percentages of compounds in EOs, without using the response factors, using gas chromatography with flame ionization detection (GC‐FID) on a Shimadzu equipment (model GC‐2010), under the same operating conditions described for the GC‐MS analysis. The essential oil analyses were performed in triplicate each replicate being an individual extraction from the same plants, indicating technical replicates.

The criterion for defining a shift in chemotype was established based on a relative percentage variation exceeding 10% for a compound to be classified as major. In accordance with ISO 11024 and ISO 4720 standards, alongside internal protocols, these benchmarks regulate the chromatographic profiles of essential oils. Consequently, a 10% delta margin is applied to secondary compounds to evaluate dominance, ultimately defining the primary chemical profile and chemotype classification of the essential oil.

### 2.3 Biological Activity—Minimum Inhibitory Concentration (MIC) and Minimum Bactericidal Concentration (MBC)

*S. aureus* (ATCC 6538) and *E. coli* (ATCC 8739) were chosen for antimicrobial evaluation. The inoculum preparation was carried out according to the recommendations of the M7‐A6 protocol for bacteria (CLSI, 2003) [23]. The cultures were incubated for 24 h in media Mueller–Hinton Broth (MHB—HiMedia, India). The bacteria were suspended in test tubes containing 4 mL of sterile saline solution. The bacterial solution was homogenized in a vortex mixer, and 2 mL aliquots were measured in a spectrophotometer at 625 nm and adjusted with 0.90% saline solution to an OD of 0.08 to 0.10, corresponding to a concentration of 1.5 × 10^8^ CFU/mL. The remaining suspensions were added with the same saline solution specifications used for the spectrophotometer adjustment. From this standard solution, serial dilutions were prepared to obtain a final concentration of 1.5 × 10^6^ CFU/mL. From this suspension, 6 mL were transferred to tubes containing 3 mL of the respective culture medium, achieving a concentration of 1 × 10^6^ CFU/mL.

The susceptibility tests were performed using the Broth Microdilution Method according to the CLSI (2005/ANVISA) Antimicrobial Susceptibility Testing Methodology. The method considers the relationship that identifies the lowest concentration that completely prevents turbidity or growth of the bacterial isolates. In 96‐well microtiter plates, each well was filled with 100 µL of MHB.

For each essential oil (EO), an aliquot of 15 mg was weighed, and the volume of culture medium required to achieve an initial in‐well concentration of 2 mg/mL was calculated. Tween 20 was added to the solutions to facilitate homogenization. A vehicle control containing Tween 20 without EOs was included to evaluate biological interference; no bacterial growth inhibition was observed, consistent with the negative control (culture medium only). A serial dilution was performed to establish a concentration gradient ranging from 2 to 0.0007 mg/mL. Following inoculation with the bacterial suspension (*A*_600_ = 0.0096), the microtiter plates were incubated at 37°C for 24 h. Postincubation, the minimum inhibitory concentrations (MICs) were determined visually using 2,3,5‐triphenyltetrazolium chloride (TTC; HiMedia, USA) as a redox indicator of metabolic activity and cell viability. Aliquots of 50 µL of a 0.1% TTC solution were added to each well, followed by a 3‐h incubation period. The assay was performed in technical triplicates. The MIC was defined as the lowest concentration that completely prevented the color transition from colorless to pink. To determine the minimum bactericidal concentration (MBC), 10 µL from wells exhibiting no visible growth were subcultured onto Mueller‐Hinton agar (MHA; HiMedia, USA) and incubated at 37°C for 24 h. The MBC was defined as the lowest concentration that resulted in no visible colony formation on the agar surface.

### 2.4 Foliar Macro and Micronutrients Quantification

Leaf samples were collected during the complete harvest cycle year (2024–2025) in the same period as the collection of samples for the EOs from representative plants corresponding to 16 from the 20 selected genotypes Four genotypes were not analyzed due to the lack of enough leaves, which were preferentially used to EOs extraction. Nutrient analyses were performed at IAC Soil Research and Development Department. Eleven nutrients were analyzed: nitrogen (N), phosphorus (P), potassium (K), calcium (Ca), magnesium (Mg), sulfur (S), iron (Fe), manganese (Mn), copper (Cu), zinc (Zn), and boron (B). The leaves were carefully cleaned to remove any adhering soil particles, washed, placed in a paper bag, and dried in a forced air oven at 65°C until constant weight. Thereafter, the samples were then weighed and grounded in a Wiley‐type mill.

A portion of the samples was used for the determination of total nitrogen (N). Dried and homogenized leaves were subjected to sulfuric digestion using the Kjeldahl method. This process, conducted with concentrated sulfuric acid (H_2_SO_4_) at high temperatures (approximately 360°C–400°C), converts organic nitrogen compounds into ammonium sulfate. Subsequently, the sample was made alkaline to distill ammonia (NH_3_), which was then quantified through colorimetric assays [24]. For the remaining nutrients Inductively Coupled Plasma Optical Emission Spectrometry (ICP‐OES) was used for simultaneous quantification method. The preparation of the samples involved acid digestion to break down the organic matrix and release the elements into a solution, which was then subjected to high temperatures (around 6.000–0.000 K), causing them to emit light at characteristic wavelengths being detected and quantified [25].

### 2.5 Climate Data

Climate data, including temperature and rainfall levels, were obtained from live measurement platforms provided by the Center for Meteorological and Climatic Research Applied to Agriculture (CEPAGRI, Campinas, SP, Brazil). Analyses were conducted using the averages recorded during the7 days prior to the harvest of plant material for EO extraction and foliar nutritional analysis.

### 2.6 Statistical Analyses

The average percentage yield of extracted essential oils was calculated as follows: (mass of essential oils obtained / mass of dry plant material) × 100, along with a heatmap, was carried out in MetaboAnalyst 6.0 using the matrix of relative area (%) for each volatile compound to illustrate the distinctions among these groups. The screening of potential antimicrobial activities was analyzed using one‐way analysis of variance (ANOVA). Posthoc comparisons of means were performed using Tukey's test against the commercial control (Chloramphenicol, MIC 0.004 mg mL^−1^) at a significance level of *p* < 0.05. Box‐and‐whisker plots were generated to highlight the distribution among the samples. For the analysis of nutrient levels, a normalization using Z‐scores was first performed, allowing comparison of nutrients of different magnitudes. This utilizes the distance of the standard deviations of a season in relation to the annual average of the evaluated nutrient. Subsequently, a repeated measures analysis of variance was performed with a significance level of *p* < 0.05. When plotted on a radar chart, the resulting diamond shape indicates that one season is statistically different from the others.

## 3 Results and Discussion

### 3.1 Onset of Seasonal and Antimicrobial Analysis of *L. alba* OEs

Leaves from 20 genotypes from IAC *L. alba* germplasm, whose chemotypes were previously characterized (Table 1), were harvested in the Spring 2021 and Summer 2021 (Sp‐21 and Su‐21, respectively) for EO extraction. As of June 2022, we identified 47 compounds. The composition varied among genotypes, confirming the existence of distinct chemotypes. Six major chemotypes were determined: myrcene, linalool, citral (neral and geranial), camphor, 1,8‐cineol, and carvone.

**TABLE 1:** Range of seasonal variation of the chemical composition from 20 genotypes of *L. alba* between different harvests years: 2021, Summer (Su‐21) and Spring (Sp‐21); 2023, Autumn (Au‐23) and Winter (Wi‐23); 2024, Spring (Sp‐24), Autumn (Au‐24), Winter (Wi‐24) and 2025, Summer (Su‐25).

Coumpound name	Summer‐2021 Su‐21	Spring ‐2021 Sp‐21	Autumn‐2023 Au‐23	Winter‐2023 Wi‐23	Summer‐2025 Su‐25	Spring‐2024 Sp‐24	Autumn‐2024 Au‐24	Winter‐2024 Wi‐24
α‐thujene (1)	0.20–0.99	0.20–0.95	0.30–0.58	0.30–6.92	—	—	—	—
α‐pinene (1)	0.31–21.27	0.19–20.75	0.41–19.87	0.38–26.30	0.92–19.59	2.46–15.79	2.42–15.07	4.27–23.64
camphene (1)	0.25–5,01	0.21–4.76	0.31–5.04	1.96–4.35	2.50–10.00	0.00–5.97	5.15–7.07	2.05–5.56
sabinene (1)	—	0.00–0.83	0.75–6.38	0.50–11.23	0.68–6.11	0.64–6.65	0.95–6.64	1.31–6.88
β‐pinene (1)	0.22–4.62	0.41–4.56	0.38–1.50	0.27–1.72	0.97–2.16	0.00–2.94	0.00–1.52	0.00–5.86
**myrcene (1)**	0.79–60.80	0.63–55.44	0.51–46.60	0.51–37.36	1.64–48.87	1.84–70.53	1.42–61.63	0.26–67.42
*n‐*octanal (4)	0.05–1.80	0.12–2.74	—	—	0.00–0.29	—	—	—
limonene (1)	0.44–32.29	0.34–31.50	0.31–28.09	0.28–3.63	0.39–16.90	1.20–18.52	1.30–22.35	2.56–15.10
**1,8‐cineole (3)**	0.21–33.38	0.52–28.57	0.66–32.66	0.41–39.86	1.89–34.63	1.46–33.50	0.84–20.78	1.86–37.53
(*E*)‐β‐ocimene (1)	0.49–2.97	0.61–2.40	0.42–1.75	0.50–1.00	0.80–2.17	—	1.73–2.32	—
**linalool (3)**	0.35–64.63	0.55–72.11	0.47–83.82	0.78–72.97	0.40–80.16	1.88–82.36	0.60–88.79	1.58–76.15
*cis*‐verbenol (3)	0.28–2.53	0.62–2.03	0.00–0.30	—	—	—	—	—
**camphor (3)**	0.26–29.01	0.32–31.77	0.27–27.79	0.87–34.93	0.75–31.37	1.64–32.93	2.29–28.40	2.34–29.44
myrtenal (3)	0.18–1.80	0.33–2.36	0.43–7.35	0.81–8.01	0.66–5.85	4.66–6.83	1.54–6.45	3.24–6.70
verbenone (3)	0.63–0.86	0.58–3.00	0.28–2.30	0.00–2.38	0.00–0.92	0.00–3.24	2.45–3.33	1.27–3.45
**neral (3)**	1.27–36.82	1.74–36.97	0.51–31.92	1.74–30.50	2.16–33.55	2.82–28.70	2.59–27.32	1.54–36.15
**carvone (3)**	2.18–44.64	2.26–48.58	49.48–50.95	4.01–58.10	5.50–63.71	17.41–65.01	61.69–63.09	3.44–65.90
**geranial (3)**	0.93–44.39	0.88–49.57	0.93–42.33	2.40–31.13	0.86–41.51	2.80–43.28	2.72–35.63	2.48–47.75
α‐ylangene (2)	0.66–6.86	0.67–6.61	0.35–1.07	1.27–6.90	0.00–1.39	—	—	0.00–2.50
β‐elemene (2)	0.45–1.94	0.45–2.07	0.52–2.10	0.85–3.45	2.49–5.88	1.11–2.63	1.53–2.73	1.75–4.14
(*Z*)‐caryophyllene (2)	3.46–6.85	—	0.00–3.15	1.20–17.53	0.00–6.70	—	0.00–4.55	0.33–18.37
(*E*)‐caryophyllene (2)	0.66–13.84	0.68–17.26	0.39–10.85	0.96–19.00	0.89–12.51	1.83–21.56	1.42–14.41	2.36–12.99
α‐guaiene (2)	0.74 ‐9.29	1.22–10.71	0.39–11.56	0,49–16.19	2.58–15.17	2.58–5.64	1.71–8.53	2.45–7.50
germacrene D (2)	0.93–14.87	0.89–6.97	0.42–20.00	1.22–11.78	0.82–16.10	2.62–12.31	0.83–23.33	1.75–25.22
*trans*‐ β ‐guaiene (2)	1.52–9.18	1.90–11.72	—	—	—	—	—	—
germacrene B (2)	0.00–3.21	—	—	—	—	—	—	—
caryophyllene oxide (5)	0.77–6.82	0.76–6.69	0.66–56.29	1.20–21,18	1.48–22.79	0.92–48.76	1.71–9.34	1.58–22.19

Chemical Classification: (1) Monoterpenes hydrocarbons, (2) Sesquiterpenes hydrocarbons, (3) Oxygen‐containing monoterpenes, (4) Others class, (5) Oxygen‐containing sesquiterpenes. Metabolites highlighted in bold represent the major compound and representative chemotype. (‐) substance not detected.

After chemotype verification, these samples were tested for their antimicrobial activity against *E. coli* and *S. aureus*. Many of these samples exhibited low MIC results indicating bacteriostatic activity (Additional Table 2). Overall, the EOs showed a stronger inhibitory response against *E.coli*. Some genotypes demonstrated a satisfactory level of inhibition with MIC values ​​below 0.062 mg/mL, such as L.a04, L.a06, L.a08, L.a10, L.a26, L.a27, L.a30, L.a36, L.a38, L.a42, L.a90, L.a129, and L.a131. However, some samples showed a variability in MIC data when comparing Sp‐21 and Su‐21. For instance, sample L.a27 in Su‐21 had a MIC of 0.031 mg/mL, while in Sp‐21 it had a MIC of 2 mg/mL. L.a38 also showed MIC differences but with a lower MIC in Sp‐21 than at Su‐21. In Su‐21, L.a89 had a MIC of 0.062 mg/mL against *E.coli*, while in Sp‐21 the same pathogen was not inhibited by the EO, indicating that a possible change of majoritarian OE could influence in MIC results. Interestingly, the L.a89 MIC against *S. aureus* did not change (Additional Table 2). Based on these results, leaves from the same plant genotypes were harvested in the autumn and winter seasons of 2023 (Au‐23 and Wi‐23) for EO extraction, in order to evaluate whether seasonality could influence EO extraction yield and chemical nature. Following the same premise, these samples were tested against *E. coli* and *S. aureus*.

### 3.2 EOs Yield and Characterization According to First Seasonal Harvesting

EOs yields ranged from 0.15% (L.a108‐Au‐23) to 1.7% (L.a118‐Su‐25) throughout the samples analyzed, with increasing diversity of constituents across seasons. On average, the highest EO yields were obtained during the summer, although some genotypes maintained relatively stable yields (Figure 1). This pattern aligns with previous reports with aromatic and medicinal species. For instance, according to Stefanakis et al. [26], summer conditions tend to increase OEs production with a higher linalool content in the Lamnaceae family (*Mentha × piperita L*. and* Mentha spicata L*). This pattern is similar to other species rich in monoterpenoids, suggesting that the rainy season tends to favor higher essential oils yields [27]. These findings support the premise that higher irradiance, elevated temperatures, and possibly higher relative humidity favor the biosynthesis and/or accumulation of volatile metabolites.

**FIGURE 1:**
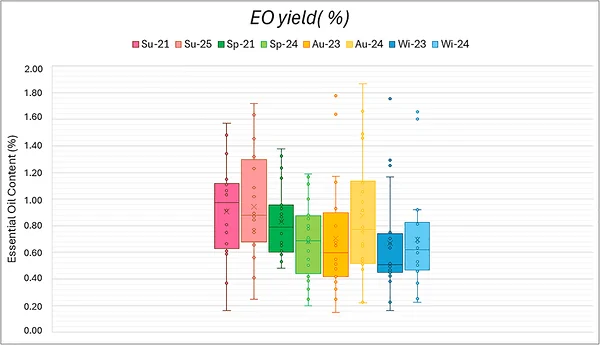
Average essential oil (EO) content of *L. alba* genotypes across harvest seasons: Summer (Su‐21) and Spring (Sp‐21) 2021; Autumn (Au‐23) and Winter (Wi‐23) 2023; Spring (Sp‐24), Autumn (Au‐24), and Winter (Wi‐24) 2024; and Summer (Su‐25) 2025.

Regarding chemotype characterization, 10 genotypes have no alteration in the six main EO compounds; consequently, its chemotype remained consistent across seasons (Figure 2; Additional Figure 1). However, for the other 10 genotypes a change in prevalent OE was detected. These modifications can be classified in four different groups. Group 1 includes genotypes L.a04, L.a22, L.a89, and L.a129, which exhibited a shift of chemotype at one of the four seasons, through the change (Figure 2; Additional Figure 1). In Group 2, the genotypes L.a30, L.a38, L.a90, and L.a105, maintain the chemotype in all the seasons but during one or two seasons the secondary compound increased resulting in a 50/50 split chemotype (Figure 2; Additional Figure 1). Group 3 comprises genotype L.a26; during transitional periods like autumn and spring, La26 shows a 50/50 division between two compounds. Interestingly, one compound is dominant in summer, while the other becomes prevalent in winter (Figure 2; Additional Figure 1). Finally, Group 4, which corresponds to L.a115, contained an average of 56%–70% of caryophyllene (*E‐*caryophyllene and caryophyllene oxide), which have emerged as significant biochemical markers of stress in aromatic plants, providing critical insights into both biotic and abiotic stress responses [28, 29]. Therefore, it is possible that L.a115 was suffering some kind of stress during harvest period.

**FIGURE 2:**
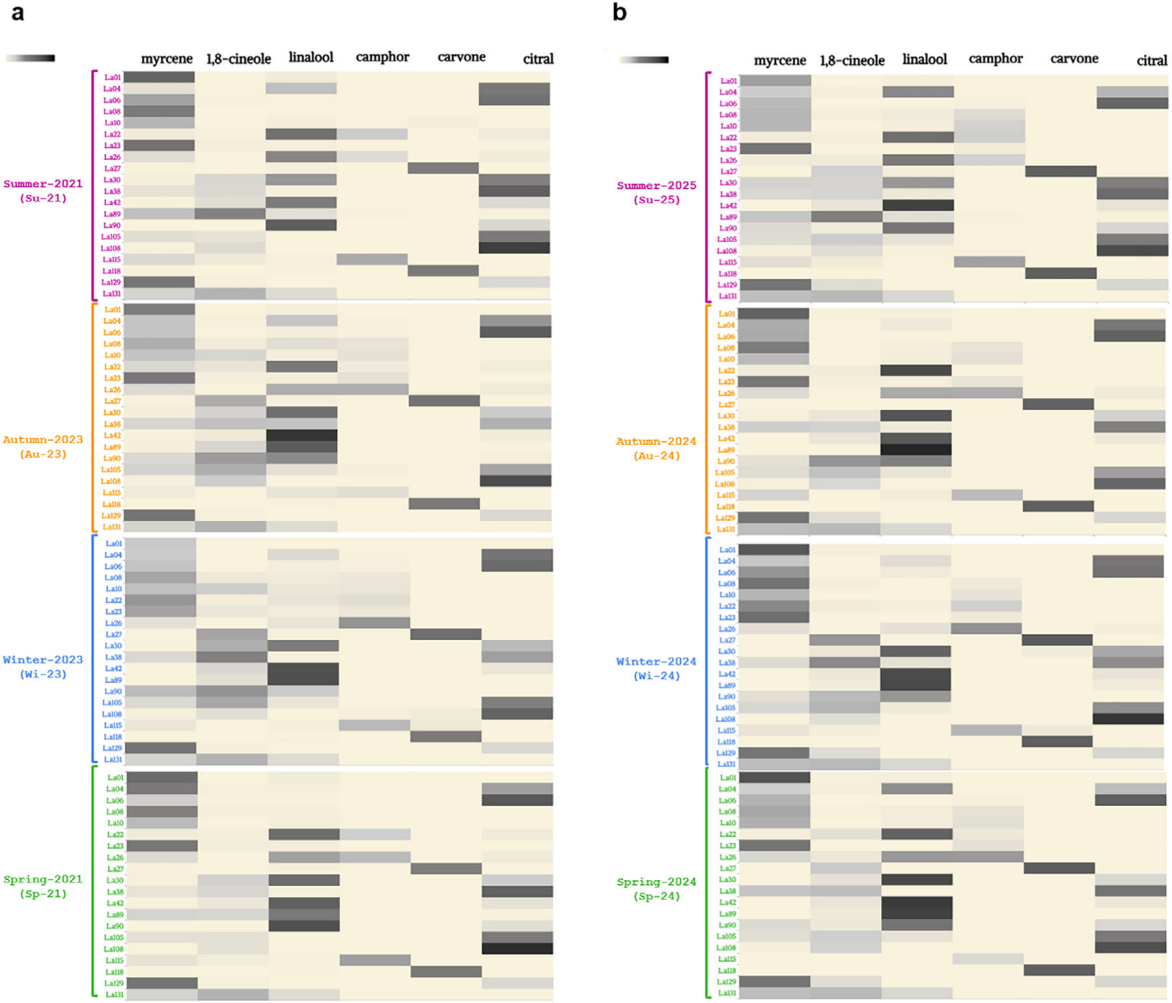
Relative abundance of six main chemical constituents identified in the EOs across different seasons. (a) First analysis using EOs from Summer of 2021 (Su‐21), Spring of 2021 (Sp‐21), Autumn of 2023 (Au‐23), and Winter of 2023 (Wi‐23). (b) Second analysis using EOs from Spring of 2024 (Sp‐24), Autumn of 2024 (Au‐24) and Winter of 2024 (Wi‐24) and Summer of 2025 (Su‐25), color intensity in the heatmap indicates the relative concentration of each compound, highlighting seasonal variations in the EO chemical profiles.

In terms of antimicrobial activity, our results showed significant variation between seasons and genotypes for both pathogens (*S. aureus and E. coli*) reinforcing the direct relationship between chemical composition and biological efficacy. The inhibitory activity of the EOs was greater against *E. coli*, with the lowest MIC value recorded at 0.008 mg/mL. On average, the lowest minimum inhibitory concentration (MIC) values were obtained from EOs extracted in summer, followed by those from spring indicating that EOs obtained in warmer seasons exhibited higher inhibitory activity against the tested microorganisms (Figure 2a). This suggests that seasonality affects not only yield and composition but also the biological efficacy of EOs.

The correlation between seasonal variation in phytochemical composition and antimicrobial effect is consistent with the literature. A study by Rocha et al. [30] revealed that hydroethanolic extracts of *L. alba* leaves exhibited marked circadian and seasonal fluctuations with higher concentrations of phenolic compounds correlating with enhanced antioxidant and antimicrobial profiles. Similarly, a geraniol‐rich chemotype of *L. alba* was identified in which EO yield and radical scavenging activity peaked during periods of lower rainfall and higher solar radiation, reinforcing the link between climatic conditions and defensive metabolite production [31].

This seasonal bioactivity relationship extends to other aromatic species. For instance, Moroccan *Lavandula mairei* Murb. showed significant seasonal variation in EO composition, with summer harvested samples containing elevated levels of 1,8‐cineole and camphor, which conferred superior antimicrobial activity compared to winter samples [32]. Likewise, *Artemisia herba‐alba* collected in the Anti‐Atlas region of Morocco exhibited maximum antifungal and antibacterial effects in October, coinciding with the highest EO yield (2.35%) and increased concentrations of camphor. Collectively, these recent investigations underscore that the antimicrobial efficacy of aromatic plant EOs is intrinsically linked to seasonal dynamics. These data reinforce that harvesting protocols must be standardized and aligned with optimal phytochemical representation to ensure reproducible therapeutic applications [33].

### 3.3 EOs Yield and Characterization According to Second Seasonal Harvesting

Another seasonal harvesting was performed between Autumn 2024 and Summer 2025 (Au‐24, Wi‐24 and Sp‐24, Su‐25) to verify whether the previously identified profiles were indeed connected to seasonal changes. Regarding EO yield, a comparison of the same seasons across different harvests showed similar yields for summer, winter, and spring. However, for the autumn samples, the yield was higher in 2024 (Au‐24) than in 2023 (Au‐23), suggesting that edaphoclimatic variations could have increased EO production in 2024 samples (Figure 1).

Strikingly, during chemotype identification, we detected that the 10 genotypes that did not exhibited seasonal chemotype modification in the 2021–2023 harvest, maintained the same profile in 2024‐2025 harvest (Figure 2, Additional Figure 1). More interestingly, the other 10 genotypes which showed seasonal chemotype modification maintained the same pattern of change detected in 2021–2023 (Figure 2, Additional Figure 1). For this second group, this seasonal phytochemical plasticity suggests that the synthesis or accumulation of specific metabolites fluctuates according to environmental conditions. The pattern observed finds parallels in studies demonstrating smooth or abrupt transitions between chemotypes throughout the annual cycle, especially in aromatic species with wide genetic variability [34, 35].

The presence of more stable and more flexible genotypes in terms of chemical composition suggests the existence of different environmental response mechanisms, possibly related to the differential expression of genes involved in monoterpene biosynthesis pathways. Such seasonal variation in chemical composition has been previously reported for *L. alba*. For example, citral chemotypes collected at different times of the year showed variation in geranial, neral, geraniol, among others, depending on the season [36]. Significant variations in the levels of citral, linalool, myrcene, and carvone are observed between the dry and rainy seasons, with a direct impact on antioxidant and antimicrobial properties [31]. Chemometric analysis showed that two distinct chemotypes exhibit considerable fluctuations throughout the year, indicating that monoterpenes (linalool and citral) biosynthesis is closely linked to local climatic conditions [37]. Furthermore, circadian studies demonstrate that even the harvest year can alter the proportions of neral, geranial, and geraniol, reinforcing the metabolic plasticity of the species [36]. Indeed, the polyploidy nature of *L. alba* and its connection to EO chemotypes suggests a complex gene expression regulation between homeologous terpene synthases (homeologous differential expression; HDE), which can be transcriptionally activated or inactivated by environmental factors, a common trait on polyploids [38, 39, 40].

Assessing the antimicrobial data, samples from Spring (Sp‐24) and Summer (Su‐25) were the most effective against the tested bacteria (Figure 3b), matching the profile found in 2021‐2023 data (Figure 3a). Nevertheless, when we compared MIC data between the two seasonal harvests, we found an overall decrease in antimicrobial activity in 24‐25 (Figure 3). This result suggests that compounds other than the major ones present in EOs can be crucial for antimicrobial activity. We hypothesize that minor components of *L. alba* are seasonally modulated and their presence or absence modifies antimicrobial activity between the two harvests. In fact, minor components alone or in combination with major compound can be responsible for the antibacterial action of EOs [41, 42].

**FIGURE 3:**
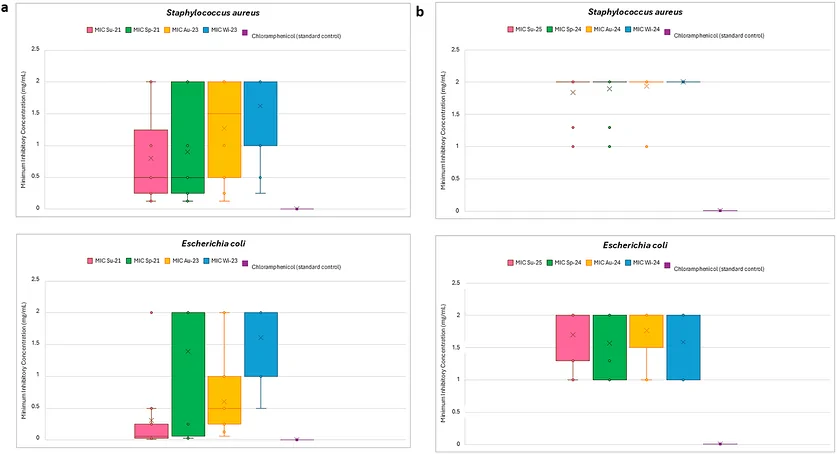
Average minimum inhibitory concentration (MIC) values of EOs against bacteria according to *L. alba* harvest seasons. ANOVA and Tukey's test with at a significance level of *p* < 0.05. (a) 2021–2023 harvest; (b) 2024–2025 harvest. *S. aureus* (upper panel) and *E. coli* (lower panel). Seasons: Summer (Su‐21) and Spring (Sp‐21) 2021; Autumn (Au‐23) and Winter (Wi‐23) 2023; Spring (Sp‐24), Autumn (Au‐24), and Winter (Wi‐24) 2024; and Summer (Su‐25) 2025.

### 3.4 Climate Data and Leaf Nutrients Evaluation

Seasonal climate variations profoundly influence the biosynthesis and accumulation of EOs in aromatic plants by modulating enzymatic activity, gene expression and precursor availability within secondary metabolic pathways. Given our finding that seasonal changes in chemotypes may modify antimicrobial effectiveness, climatic data throughout our experiments were collected and evaluated. For summer, spring and winter samples, we did not detect significant differences in rainfall or mean temperature, when comparing the 2021–2023 and 2024–2025 harvests (Figure 4). Nevertheless, in Au‐23 rainfall twice as high comparing to Au‐24 (Figure 4), which may correlate with the difference in OE yield between these harvests (Figure 1). Studies confirm that these climatic factors directly alter the qualitative and quantitative profiles of terpenoids and phenylpropanoids in EOs. For example, research on *Eucalyptus microcorys* demonstrated that canonical redundancy analysis could correlate monthly variations in essential oil composition with rainfall, humidity, and mean temperature, revealing strong associations between specific monoterpenes and phenolic compounds [43].

**FIGURE 4:**
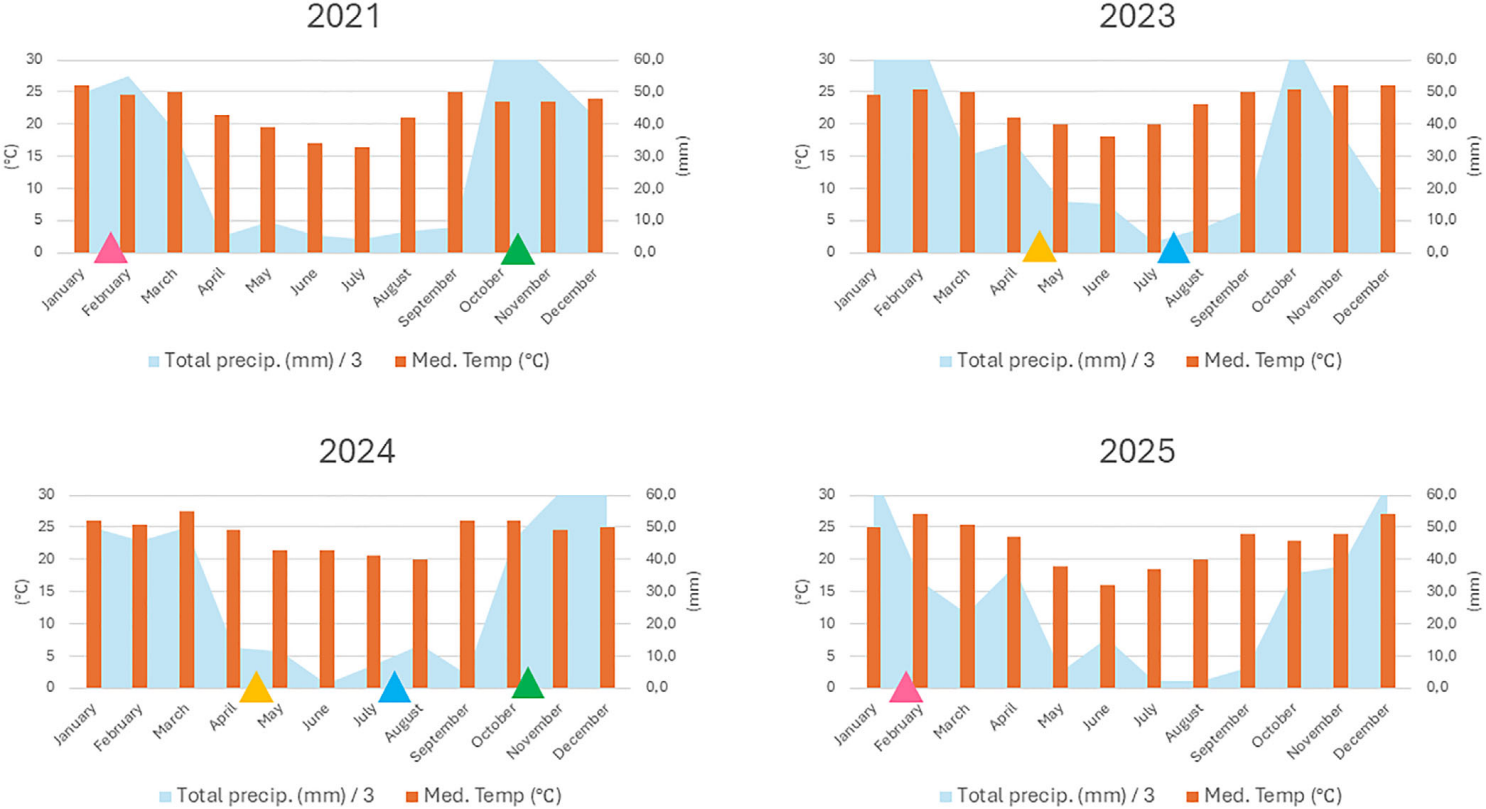
Mean weather conditions during the growth cycles across the four harvest years. Triangles represent the harvests by season: Summer (pink: Su‐21 and Su‐25), Spring (green: Sp‐21 and Sp‐24), Autumn (orange: Au‐23 and Au‐24), and Winter (blue: Wi‐23 and Wi‐24).

Additionally, soil and leaf nutrient status serves as a foundational determinant of phytochemical output, as shown in *Siparuna muricata*, a medicinal plant from Ecuador [44]. Therefore, we evaluated the leaf nutrient content of our *L. alba* germplasm. Although insufficient plant material was available for leaf nutrient analysis during the 2021–2023 harvests, we were able to evaluate macro‐ and micronutrients in the leaves of 16 out of 20 genotypes from the 2024–2025 period. Our analysis revealed that the availability of essential macronutrients (nitrogen, phosphorus, and potassium) is not dependent on seasonality, with only minor fluctuations occurring during the autumn and spring seasons (Additional Figure 2).

Regarding micronutrients, the values found in *L. alba* genotypes were, on average, below 100 ppm for the summer and spring seasons (Figure 5), with the exception of iron (Fe) levels. Notably, genotype La108 (Sp‐24) showed values above 600 ppm. Interestingly, according to Yadegari [45], the aromatic plant Damask rose treated with a maximum of 400 ppm of zinc, iron, manganese, and copper (Zn, Fe, Mn, and Cu, respectively), particularly at 40 ppm, showed an increase in EO content. Hence, we decided to assess the contents of these micronutrients in the three groups exhibiting seasonal chemotype changes.

**FIGURE 5:**
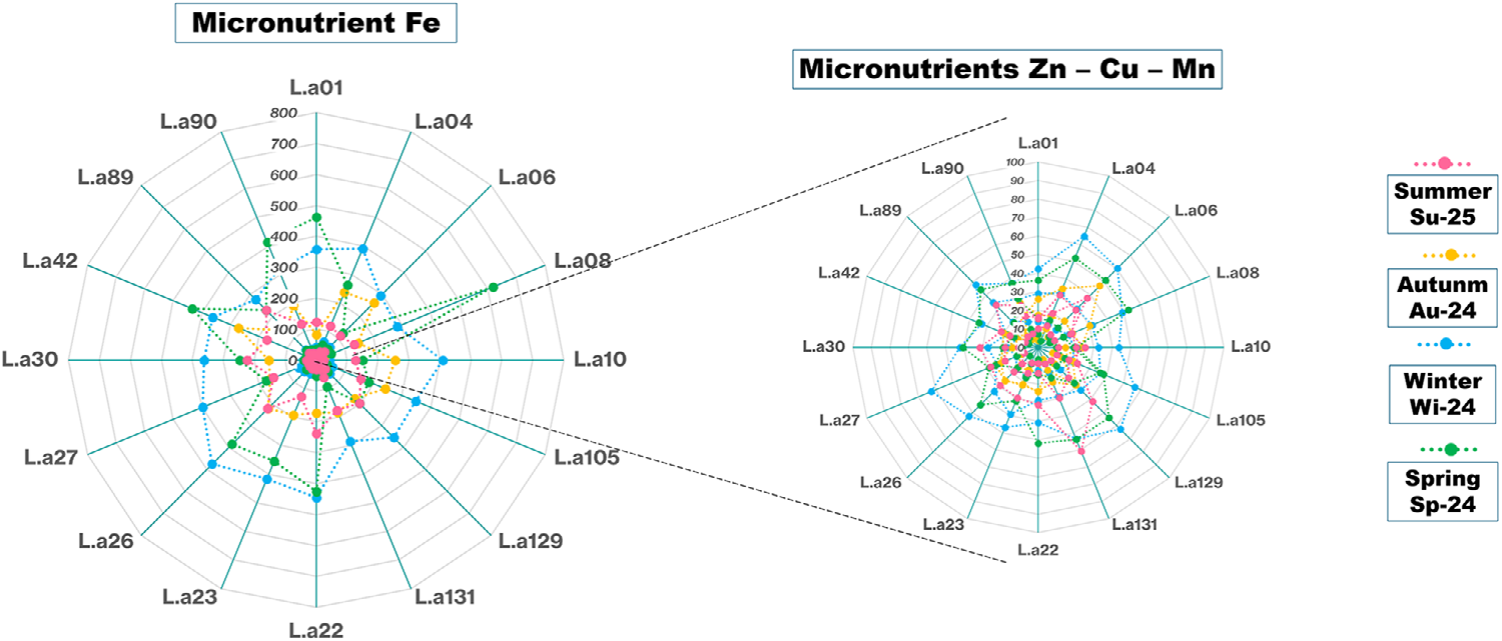
Foliar micronutrient content (Zn, Cu, Mn, and Fe) in representative germplasm genotypes according to harvest time. Z‐scores were applied. Radar charts indicate micronutrient concentrations in ppm.

Our results showed higher levels of Mn during the 2024 spring season, a behavior shared among the genotypes, especially among those belonging to groups that undergo alterations in their chemotype profiles between seasons (Figure 6). Laftouhi et al., [46] indicated that cooler spring temperatures generally enhance the solubility and root uptake of Mn from the soil, a period that often coincides with peak production of phenolic compounds and monoterpenes. Copper is essential for the activity of plastocyanin and polyphenol oxidase (PPO), an enzyme directly involved in phenolic metabolism [47]. Although the availability and plant demand for zinc and copper are profoundly influenced by seasonal dynamics, our data did not reveal a consistent pattern among all samples, indicating that genotypic particularities exert a greater effect on their presence or absence (Figure 6a,b). However, applications of Zinc in autumn have been shown to enhance the quality of essential oils in *Lavandula angustifolia* by upregulating geraniol synthase [48]. Such data corroborate our findings, in which genotypes La90 and La129 (which undergo profile changes) exhibited an increase in Zn concentration during the Au‐24 period compared to the other genotypes (Figure 6a,b).

**FIGURE 6:**
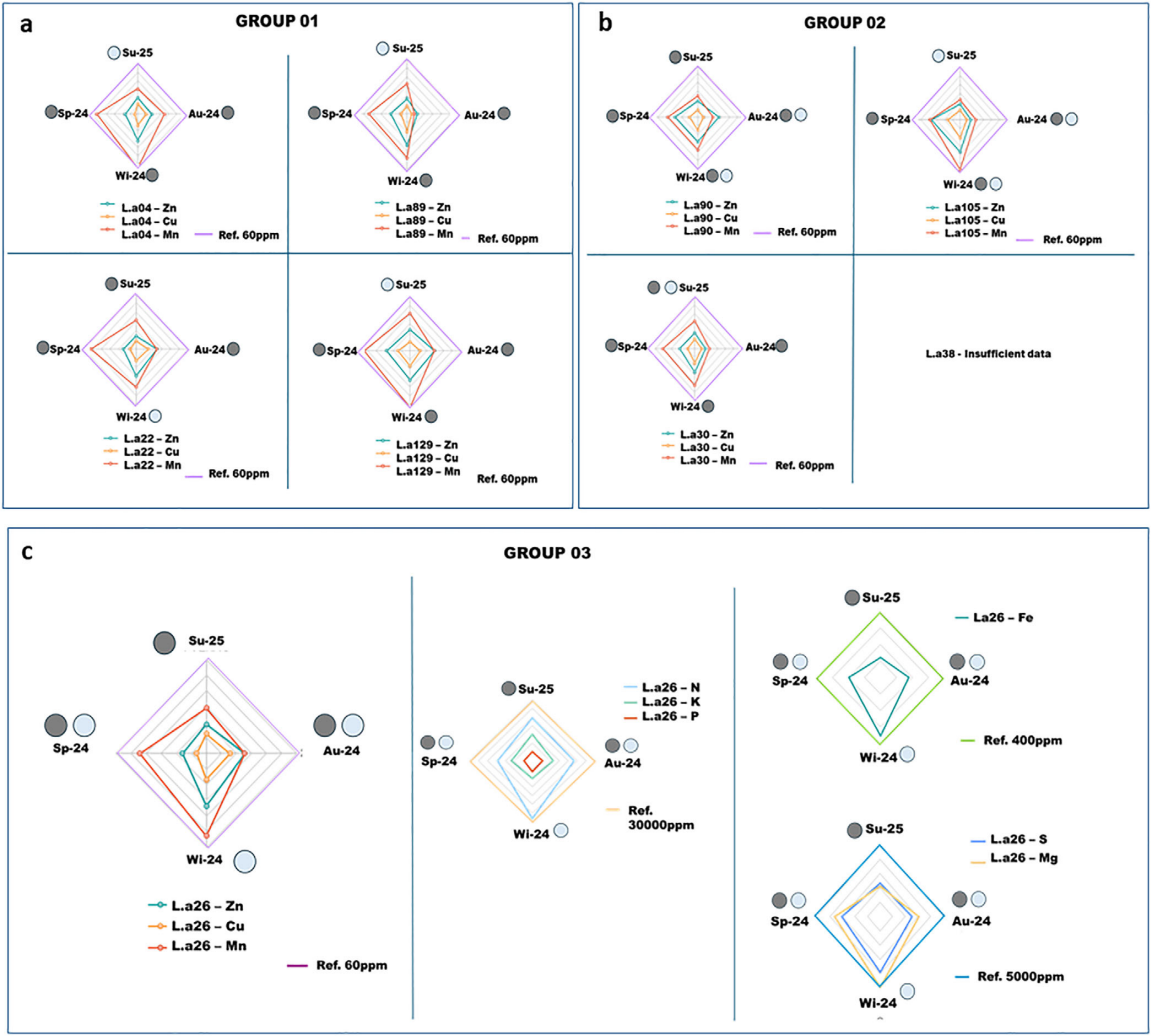
Nutrient levels in genotypes exhibiting seasonal shifts in chemical composition. Data are presented in adapted diamond‐shaped radar charts. Z‐scores were applied and repeated measures analysis of variance was performed with a significance level of *p* < 0.05. Different colored circles above each season indicate compound shifts. (a) Concentration of Zn, Cu, and Mn in Group 1 genotypes (major compound shifts between seasons); reference value = 60 ppm. (b) Concentration of Zn, Cu, and Mn in Group 2 genotypes (major compound equivalent to secondary compound); reference value = 60 ppm. (c) Nutrient content in Group 3 genotypes (cyclical seasonal compound shifts). Left: Zn, Cu, and Mn levels; Center: Macronutrients N, P, and K (reference value = 30,000 ppm); Upper right: Fe levels (reference value = 400 ppm); Lower right: S and Mg levels (reference value = 5,000 ppm).

Iron is a central player, acting as a critical cofactor for phenylalanine ammonia‐lyase (PAL)—the gateway enzyme to the phenylpropanoid pathway—as well as for various cytochrome P450 monooxygenases and peroxidases involved in the synthesis of lignin, flavonoids, and volatile oils [49]. Our results showed that the La26 genotype (Group 3) exhibited a reduction in Fe levels in Su‐25 and an increase in Wi‐24 (Figure 6c), correlating with the major changes in its chemical profile. Notably, La26 presented MIC values of 0.125 mg/mL (*S. aureus*) and 0.031 mg/mL (*E. coli*) during Su‐25, but showed no inhibition during Wi‐24, indicating a possible correlation between iron, seasonal chemotype modifications, and antibacterial activity. Regarding the La26 genotype, the same trend observed between Su‐25 and Wi‐24 applies to sulfur and magnesium, with concentrations increasing during the winter (Figure 6c). In contrast, the macronutrient potassium (K) exhibited an opposite trend, increasing in the summer and decreasing in the winter (Figure 6c). These stresses trigger defensive responses that often involve Cu/Zn‐SOD, diverting metabolic resources toward the synthesis of protective secondary metabolites [50].

We cannot rule out the possibility that other environmental factors not evaluated in this study, such as soil composition and microbiota diversity, may also influence secondary metabolite synthesis. Plant age represents another relevant variable; because the same plants were monitored across consecutive seasonal harvests, the specimens were inherently older during the second harvest cycle. Although data from Eucalyptus globulus indicate that EOs from both young and adult leaves exhibit similar herbicidal, antibacterial, and insecticidal activities, quantitative variations have been detected in their chemical compositions [51]. Furthermore, the potential contribution of minor compounds within the EOs and their synergistic interactions with major constituents warrant further investigation [41]. This could be addressed through absolute quantification studies, the isolation of individual compounds, and biological activity assays utilizing pure constituents.

## 4 Conclusion

Our data suggest that *L. alba* essential oils can be influenced by a complex interaction between genotype and harvest season. Even though, our data are exploratory, since only one plant for each genotype and season was evaluated, the identification of key compounds, the seasonal shifts in major constituents within specific genotypes, and the low MIC values observed in summer/spring EOs point to the importance of considering both genetics and environmental conditions when developing botanical, phytotherapeutic, or industrial applications for *L. alba*. The existence of genotypes with unstable chemical behavior throughout the year highlights the need for rigorous monitoring in commercial cultivation systems, especially to ensure consistency between production batches. Expanding this perspective will not only provide a deeper understanding of the mechanisms regulating terpene biosynthesis in *L. alba* but also guide management and genetic improvement strategies aimed at developing more productive, stable genotypes with higher bioactive compound contents.

## Authors Contributions

M.E.A.H.P.S. performed the essential oil extractions, assisted with the antimicrobial experiments, conducted the statistical analysis, and drafted the manuscript. T.G.B. assisted with the essential oil extractions and antimicrobial experiments. C.D. performed the antimicrobial experiments. W.J.S. was coresponsible for *L. alba* germplasm maintenance. M.C.T.D. supervised and coordinated the antimicrobial analyses. M.O.M.M. was coresponsible for *L. alba* germplasm maintenance and coordinated the chemical composition analyses of the essential oils. J.M.C.M. conceptualized the study, assisted with the statistical analysis, and was responsible for drafting and finalizing the manuscript.

## Funding

This research was supported by the São Paulo Research Foundation (FAPESP, grants 2017/50338‐9 and 2018/25812‐1). M.O.M.M. is grateful to the National Council for Scientific and Technological Development (CNPq) for a productivity fellowship (grant 07929/2022‐1). M.E.A.H.P.S. was supported by a doctoral fellowship from FAPESP (grant 2023/03799‐1), and T.G.B. by a master's fellowship from the Coordination for the Improvement of Higher Education Personnel (CAPES).

## Conflicts of Interest

The authors declare no conflicts of interest.

## Supporting information




**Supporting File 1**: cbdv71745‐sup‐0001‐SuppMat.docx.

## Data Availability

Data is available upon request to the authors.
