# Supplementary material for: Seasonal Variation of Essential Oils Composition in a Genetically Diverse Population of Lippia alba and Its Influence on Antibacterial Activity
Source: Chem Biodivers. 2026 Oct 6;23(10):e71745. doi: 10.1002/cbdv.71745 (PMC13640040; doi:10.1002/cbdv.71745)
Supplement: Supplementary file 1 [file CBDV-23-e71745-s001.docx]

**SUPPORTING INFORMATION**

**Additional Table 1:** List of preselected *Lippia alba* genotypes from the germplasm bank. Corresponding chemotypes, seasonal shifts groups to which they belong, and codes for the harvest years.

| **Genotypes** | **Chemotype** | **Group*** | **Harvest period** | | | | | | | |
| --- | --- | --- | --- | --- | --- | --- | --- | --- | --- | --- |
|  |  |  | **Summer-2021**  **Su-21** | **Spring -2021**  **Sp-21** | **Autumn-2023**  **Au-23** | **Winter-2023**  **Wi-23** | **Summer-2025**  **Su-25** | **Spring -2024**  **Sp-24** | **Autumn-2024**  **Au-24** | **Winter-2024**  **Wi-24** |
| **L.a01** | myrcene | - | L.a01-Su21 | L.a01-Sp21 | L.a01-Au23 | L.a01-Wi23 | L.a01-Su25 | L.a01-Sp24 | L.a01-Au24 | L.a01-Wi24 |
| **L.a04** | citral | 1 | L.a04-Su21 | L.a04-Sp21 | L.a04-Au23 | L.a04-Wi23 | L.a04-Su25 | L.a04-Sp24 | L.a04-Au24 | L.a04-Wi24 |
| **L.a06** | citral | - | L.a06-Su21 | L.a06-Sp21 | L.a06-Au23 | L.a06-Wi23 | L.a06-Su25 | L.a06-Sp24 | L.a06-Au24 | L.a06-Wi24 |
| **L.a08** | myrcene | - | L.a08-Su21 | L.a08-Sp21 | L.a08-Au23 | L.a08-Wi23 | L.a08-Su25 | L.a08-Sp24 | L.a08-Au24 | L.a08-Wi24 |
| **L.a10** | myrcene | - | L.a10-Su21 | L.a10-Sp21 | L.a10-Au23 | L.a10-Wi23 | L.a10-Su25 | L.a10-Sp24 | L.a10-Au24 | L.a10-Wi24 |
| **L.a22** | linalool | 1 | L.a22-Su21 | L.a22-Sp21 | L.a22-Au23 | L.a22-Wi23 | L.a22-Su25 | L.a22-Sp24 | L.a22-Au24 | L.a22-Wi24 |
| **L.a23** | myrcene | - | L.a23-Su21 | L.a23-Sp21 | L.a23-Au23 | L.a23-Wi23 | L.a23-Su25 | L.a23-Sp24 | L.a23-Au24 | L.a23-Wi24 |
| **L.a26** | linalool-camphor | 3 | L.a26-Su21 | L.a26-Sp21 | L.a26-Au23 | L.a26-Wi23 | L.a26-Su25 | L.a26-Sp24 | L.a26-Au24 | L.a26-Wi24 |
| **L.a27** | carvone | - | L.a27-Su21 | L.a27-Sp21 | L.a27-Au23 | L.a27-Wi23 | L.a27-Su25 | L.a27-Sp24 | L.a27-Au24 | L.a27-Wi24 |
| **L.a30** | linalool | 2 | L.a30-Su21 | L.a30-Sp21 | L.a30-Au23 | L.a30-Wi23 | L.a30-Su25 | L.a30-Sp24 | L.a30-Au24 | L.a30-Wi24 |
| **L.a38** | citral | 2 | L.a38-Su21 | L.a38-Sp21 | L.a38-Au23 | L.a38-Wi23 | L.a38-Su25 | L.a38-Sp24 | L.a38-Au24 | L.a38-Wi24 |
| **La.42** | linalool | - | La.42-Su21 | La.42-Sp21 | La.42-Au23 | La.42-Wi23 | La.42-Su25 | La.42-Sp24 | La.42-Au24 | La.42-Wi24 |
| **L.a89** | linalool | 1 | L.a89-Su21 | L.a89-Sp21 | L.a89-Au23 | L.a89-Wi23 | L.a89-Su25 | L.a89-Sp24 | L.a89-Au24 | L.a89-Wi24 |
| **L.a90** | linalool | 2 | L.a90-Su21 | L.a90-Sp21 | L.a90-Au23 | L.a90-Wi23 | L.a90-Su25 | L.a90-Sp24 | L.a90-Au24 | L.a90-Wi24 |
| **L.a105** | citral | 2 | L.a105-Su21 | L.a105-Sp21 | L.a105-Au23 | L.a105-Wi23 | L.a105-Su25 | L.a105-Sp24 | L.a105-Au24 | L.a105-Wi24 |
| **L.a108** | citral | - | L.a108-Su21 | L.a108-Sp21 | L.a108-Au23 | L.a108-Wi23 | L.a108-Su25 | L.a108-Sp24 | L.a108-Au24 | L.a108-Wi24 |
| **L.a115** | camphor | 4 | L.a115-Su21 | L.a115-Sp21 | L.a115-Au23 | L.a115-Wi23 | L.a115-Su25 | L.a115-Sp24 | L.a115-Au24 | L.a115-Wi24 |
| **L.a118** | carvone | - | L.a118-Su21 | L.a118-Sp21 | L.a118-Au23 | L.a118-Wi23 | L.a118-Su25 | L.a118-Sp24 | L.a118-Au24 | L.a118-Wi24 |
| **L.a129** | citral | 1 | L.a129-Su21 | L.a129-Sp21 | L.a129-Au23 | L.a129-Wi23 | L.a129-Su25 | L.a129-Sp24 | L.a129-Au24 | L.a129-Wi24 |
| **L.a131** | myrcene-eucalyptol | - | L.a131-Su21 | L.a131-Sp21 | L.a131-Au23 | L.a131-Wi23 | L.a131-Su25 | L.a131-Sp24 | L.a131-Au24 | L.a131-Wi24 |

|  | ***Staphylococcus aureus*** | | | | | | | | | | | | | | | | |
| --- | --- | --- | --- | --- | --- | --- | --- | --- | --- | --- | --- | --- | --- | --- | --- | --- | --- |
|  | **MIC Su-21** | **MBC Su-21** | **MIC Sp-21** | **MBC Sp-21** | **MIC Au-23** | **MBC Au-23** | **MIC Wi-23** | **MBC Wi-23** |  | **MIC Su-25** | **MBC Su-25** | **MIC Sp-24** | **MBC Sp-24** | **MIC Au-24** | **MBC Au-24** | **MIC Wi-24** | **MBC Wi-24** |
| **L.a01** | Y | N | Y | N | Y | Y | Y | N |  | N | N | N | N | N | N | N | N |
| **L.a04** | Y | Y | Y | Y | Y | Y | Y | Y |  | N | N | N | N | N | N | N | N |
| **L.a06** | Y | Y | Y | Y | Y | Y | Y | Y |  | N | N | N | N | N | N | N | N |
| **L.a08** | Y | Y | Y | Y | Y | N | N | N |  | N | N | N | N | N | N | N | N |
| **L.a10** | Y | Y | Y | Y | Y | N | N | N |  | N | N | N | N | N | N | N | N |
| **L.a22** | N | N | N | N | Y | N | N | N |  | N | N | N | N | N | N | N | N |
| **L.a23** | N | N | N | N | N | N | N | N |  | N | N | N | N | N | N | N | N |
| **L.a26** | Y | Y | Y | Y | Y | N | N | N |  | N | N | N | N | N | N | N | N |
| **L.a27** | Y | Y | Y | Y | Y | N | N | N |  | N | N | N | N | N | N | N | N |
| **L.a30** | Y | Y | Y | Y | Y | N | Y | N |  | N | N | N | N | N | N | N | N |
| **L.a38** | Y | Y | N | N | Y | Y | Y | Y |  | N | N | N | N | Y | N | N | N |
| **La.42** | Y | Y | Y | Y | N | N | N | N |  | Y | Y | N | N | N | N | N | N |
| **L.a89** | Y | N | Y | N | Y | N | Y | N |  | N | N | N | N | N | N | N | N |
| **L.a90** | Y | Y | Y | Y | Y | Y | N | N |  | N | N | N | N | N | N | N | N |
| **L.a105** | Y | Y | Y | Y | Y | Y | Y | Y |  | N | N | N | N | N | N | N | N |
| **L.a108** | N | N | N | N | Y | Y | N | N |  | Y | Y | Y | Y | N | N | N | N |
| **L.a115** | N | N | N | N | N | N | N | N |  | N | N | N | N | N | N | N | N |
| **L.a118** | Y | Y | Y | Y | N | N | N | N |  | Y | Y | N | N | N | N | N | N |
| **L.a129** | Y | Y | Y | Y | Y | Y | Y | N |  | N | N | Y | Y | N | N | N | N |
| **L.a131** | Y | Y | Y | Y | Y | Y | Y | Y |  | N | N | N | N | N | N | N | N |
|  | ***Escherichia coli*** | | | | | | | | | | | | | | | | |
|  | **MIC Su-21** | **MBC Su-21** | **MIC Sp-21** | **MBC Sp-21** | **MIC Au-23** | **MBC Au-23** | **MIC Wi-23** | **MBC Wi-23** |  | **MIC Su-25** | **MBC Su-25** | **MIC Sp-24** | **MBC Sp-24** | **MIC Au-24** | **MBC Au-24** | **MIC Wi-24** | **MBC Wi-24** |
| **L.a01** | Y | Y | N | N | Y | Y | Y | Y |  | N | N | N | N | N | N | N | N |
| **L.a04** | Y | Y | Y | Y | Y | Y | Y | Y |  | N | N | N | N | N | N | N | N |
| **L.a06** | Y | Y | N | N | Y | Y | Y | Y |  | N | N | N | N | N | N | N | N |
| **L.a08** | Y | Y | N | N | Y | Y | Y | Y |  | N | N | N | N | N | N | N | N |
| **L.a10** | Y | Y | N | N | Y | Y | Y | Y |  | N | N | N | N | N | N | N | N |
| **L.a22** | N | N | Y | Y | Y | Y | N | N |  | N | N | N | N | N | N | N | N |
| **L.a23** | Y | Y | N | N | N | N | N | N |  | N | N | N | N | N | N | N | N |
| **L.a26** | Y | Y | Y | Y | Y | Y | N | N |  | N | N | N | N | N | N | N | N |
| **L.a27** | Y | Y | N | N | Y | Y | Y | Y |  | Y | N | Y | N | Y | N | Y | N |
| **L.a30** | Y | Y | N | N | Y | Y | Y | Y |  | N | N | Y | N | N | N | Y | N |
| **L.a38** | Y | Y | Y | Y | Y | Y | Y | Y |  | Y | Y | Y | Y | N | N | N | N |
| **La.42** | Y | Y | N | N | N | N | Y | N |  | N | N | N | N | N | N | Y | N |
| **L.a89** | Y | Y | N | N | Y | Y | N | N |  | N | N | N | N | N | N | N | N |
| **L.a90** | Y | Y | Y | Y | Y | Y | Y | Y |  | Y | N | Y | N | Y | N | Y | N |
| **L.a105** | Y | Y | N | N | Y | Y | Y | Y |  | N | N | N | N | N | N | N | N |
| **L.a108** | N | N | Y | Y | Y | Y | N | N |  | Y | N | Y | N | N | N | Y | N |
| **L.a115** | N | N | Y | Y | Y |  | Y | Y |  | N | N | N | N | N | N | N | N |
| **L.a118** | Y | Y | N | N | Y | Y | N | N |  | Y | Y | N | N | N | N | N | N |
| **L.a129** | Y | Y | Y | Y | Y | Y | Y | Y |  | Y | N | Y | N | Y | N | Y | N |
| **L.a131** | Y | Y | N | N | Y | Y | Y | Y |  | N | N | Y | N | N | N | N | N |

**Additional Table 2**. Presence (Y) or absence (N) of antimicrobial activity based on minimum inhibitory concentration (MIC) and minimum bactericidal concentration (MBC) with essential oils from different harvest years: 2021, Summer (Su-21) and Spring (Sp21); 2023, Autumn (Au-23) and Winter (Wi-23); 2024, Spring (Sp-24), Autumn (Au-24) and Winter (Wi-24) and 2025 Summer (Su-25). Values below 2mg/mL were considered acceptable for cutoff of inhibitory activity.

**Additional Figure 1**: Relative abundance of chemical constituents identified in the EOs across different seasons. Upper panel: Summer (Su-21) and Spring (Sp-21) 2021; Autumn (Au-23) and Winter (Wi-23) 2023. Lower panel: Spring (Sp-24), Autumn (Au-24), and Winter (Wi-24) 2024; and Summer (Su-25) 2025. Color intensity in the heatmap indicates the relative concentration of each compound, highlighting seasonal variations in the EO chemical profiles.

**Additional figure 2**: Availability of macronutrients (N-nitrogen, P-phosphorus and K-potassium) to the 14 representative genotypes of the germplasm and arranged according to the season harvest: 2024, Spring (Sp24), Autumn (Au-24) and Winter (Wi-24); and 2025 Summer (Su-25).
